# CD147 knockdown improves the antitumor efficacy of trastuzumab in HER2-positive breast cancer cells

**DOI:** 10.18632/oncotarget.10252

**Published:** 2016-06-23

**Authors:** Lijuan Xiong, Li Ding, Haoyong Ning, Chenglin Wu, Kaifei Fu, Yuxiao Wang, Yan Zhang, Yan Liu, Lijun Zhou

**Affiliations:** ^1^ Central Laboratory, Navy General Hospital, Beijing 100048, P.R. China; ^2^ The Third School of Clinical Medicine, Southern Medical University, Guangzhou, Guangdong, 510630, P.R.China; ^3^ Department of Pathology, Navy General Hospital, Beijing 100048, P.R. China; ^4^ Department of Surgery, Navy General Hospital, Beijing 100048, P.R. China

**Keywords:** CD147, HER2, breast cancer, antibody drug resistance/sensitivity, trastuzumab efficacy

## Abstract

Trastuzumab is widely used in the clinical treatment of human epidermal growth factor receptor-2 (HER2)-positive breast cancer, but the patient response rate is low. CD147 stimulates cancer cell proliferation, migration, metastasis and differentiation and is involved in chemoresistance in many types of cancer cells. Whether CD147 alters the effect of trastuzumab on HER2-positive breast cancer cells has not been previously reported. Our study confirmed that CD147 suppression enhances the effects of trastuzumab both *in vitro* and *in vivo*. CD147 suppression increased the inhibitory rate of trastuzumab and cell apoptosis in SKBR3, BT474, HCC1954 and MDA-MB453 cells compared with the controls. Furthermore, CD147 knockdown increased expression of cleaved Caspase-3/9 and poly (ADP-ribose) polymerase (PARP) and decreased both mitogen-activated protein kinase (MAPK) and Akt phosphorylation in the four cell lines. In an HCC1954 xenograft model, trastuzumab achieved greater suppression of tumor growth in the CD147-knockdown group than in the shRNA negative control (NC) group. These data indicated that enhancement of the effect of trastuzumab on HER2-positive cells following CD147 knockdown might be attributed to increased apoptosis and decreased phosphorylation of signaling proteins. CD147 may be a key protein for enhancing the clinical efficacy of trastuzumab.

## INTRODUCTION

Breast cancer is one of the major malignancies threatening women's health. Worldwide, approximately 1.2 million new cases of breast cancer are diagnosed, and approximately 500,000 women die of breast cancer each year. HER2 (ErbB2 or NEU), a member of the ErbB family of receptor tyrosine kinases (RTKs), is the main pathogenic gene in breast cancer; HER2 overexpression has been demonstrated in 20 to 30% of patients [1, 2]. Trastuzumab (Herceptin), a humanized monoclonal antibody against HER2 developed by Genentech, was approved by the Food and Drug Administration (FDA) for the treatment high HER2-expression breast cancer in 1998. However, the objective response rate to trastuzumab is only 30%, and most breast cancer patients with high HER2 expression do not respond to trastuzumab treatment [3, 4]. Hence, efforts to improve drug treatment efficacy have received widespread attention. The goals of these efforts have included elucidation of the underlying mechanisms [4], identification of the synergistic effects of new target proteins [5], and the construction of bispecific or multi-specific antibodies.

CD147, or HAb18G, a transmembrane glycoprotein, is a member of the human immunoglobulin superfamily [6–8]. It promotes tumor invasion, growth and metastasis by stimulating matrix metalloproteinase (MMP) synthesis in neighboring fibroblasts [9] and inhibiting cell apoptosis [10]. In addition, it promotes chemoresistance in different cancer cell types, including breast cancer cells [11–15], by stimulating hyaluronan production [16]. In hepatocellular carcinoma (HCC) and breast cancer, CD147 increases multi-drug resistance-1 and MMP-11 expression [12, 17–19]. Moreover, CD147 correlates with epidermal growth factor receptor activity in association with tumor chemoresistance in a cancer stem cell-like cell line derived from breast cancer cells [20]. However, wheher CD147 is related to antibody drug efficacy has not yet been elucidated.

In this study, we investigated whether CD147 alters the efficacy of trastuzumab against HER2-positive cancer in both trastuzumab-sensitive and -resistant breast cancer cell lines via CD147 knockdown and preliminarily explored the underlying mechanism. We also assessed the effect of CD147 on the antitumor efficacy of trastuzumab in a breast cancer xenograft model. Our results may provide insights for improving the efficacy of trastuzumab in HER2-positive breast cancer patients.

## RESULTS

### Verification of CD147 and HER2 expression *in vivo*

**Figure 1:**
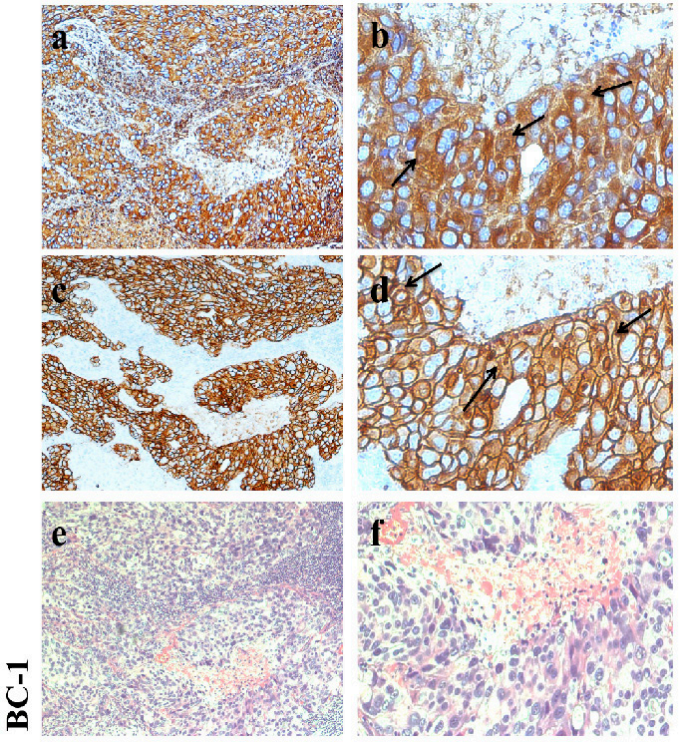
Immunohistochemical analysis of CD147 and HER2 expression in HER2-positive breast cancer **a, b.** CD147 expression in breast cancer. **c, d.** HER2 expression in breast cancer. **e, f.** Hematoxylin-eosin (HE) staining. Left: 100× magnification; and right: 400× magnification.

CD147 is expressed at a high level in cancer tissues and at a low level in normal tissues [21]. CD147 and HER2 expression in 271 breast cancer samples, as demonstrated by streptavidin-alkaline phosphatase immunohistochemistry (S-P IHC) analysis, has been previously reported [22, 23]. Here, we also detected CD147 and HER2 expression in 10 clinical breast cancer tissue samples (including 9 HER2-positive samples) by IHC to provide a foundation for subsequent analyses. The staining results for one sample are presented in Figure 1 (the rest of the results are shown in Supplementary Figure S1). CD147 and HER2 were mainly expressed on membranes. According to our results, CD147 was highly expressed in HER2-positive breast cancer tissues, verifying that CD147 and HER2 are frequently simultaneously expressed in HER2-positive breast cancer tissues *in vivo*.

### Verification of CD147 and HER2 expression in HER2-positive cancer cells

**Figure 2:**
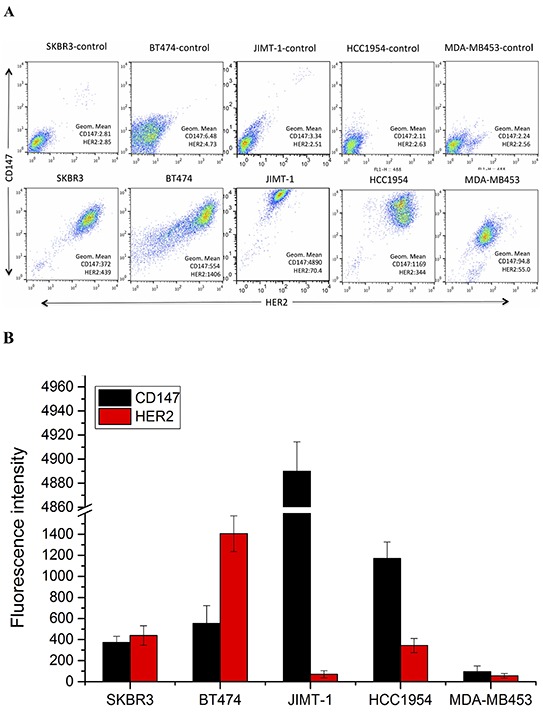

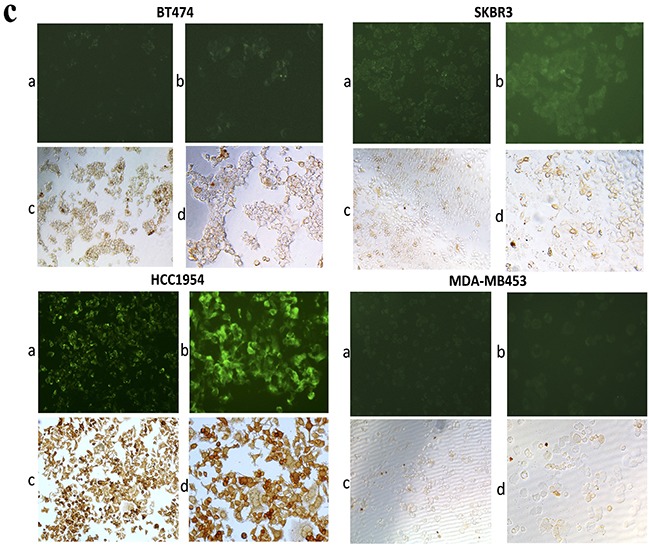
CD147 and HER2 expression in different HER2-positive cancer cell lines **A.** and **B.** CD147 and HER2 protein expression in cancer cells, as measured by FACS. **C.** CD147 protein expression in BT474, SKBR3, HCC1954 and MDA-MB453 cells; a and b were detected by IF under 100× and 200× magnification; and c and d were detected by ICC under 100× and 200× magnification.

To further confirm the IHC results, we performed fluorescence-activated cell sorting (FACS) to analyze CD147 and HER2 expression in different HER2-positive breast cancer cell lines (Figure 2A, 2B). High CD147 expression and low HER2 expression were observed in JIMT-1 and HCC1954 cells, and moderate expression of both CD147 and HER2 was detected in SKBR3 cells. Further, moderate CD147 expression and high HER2 expression were observed in BT474 cells, and low expression of both CD147 and HER2 was found in MDA-MB453 cells. We also detected CD147 expression in cancer cells by immunofluorescence (IF) and immunocytochemistry (ICC) (Figure 2C), demonstrating similar results as those described above in the four cell lines. According to the literature, HCC1954 and MDA-MB453 are always regarded as trastuzumab-resistant cell lines, and BT474 and SKBR3 are regarded as trastuzumab-sensitive cell lines. Therefore, we chose these four cell lines for further study.

### CD147 expression is decreased by specific siRNAs in HER2-positive breast cancer cells

**Figure 3:**
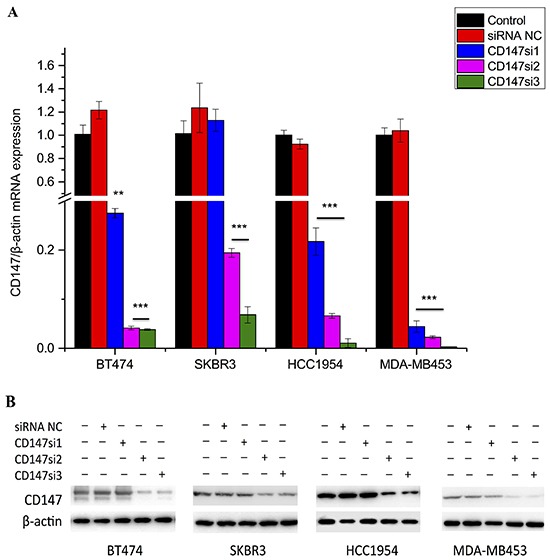
CD147 mRNA and protein expression levels were reduced by specific siRNAs **A.** CD147 mRNA expression was detected by real-time PCR in different cell lines; β-actin was used as a normalization control. Inhibition of greater than 80% was regarded as effective siRNA interference. *p<0.05, **p<0.01 and ***p<0.001 compared with control. **B.** CD147 protein expression was detected by Western blotting in different cancer cells.

The effects of specific siRNAs (Table 1) on CD147 expression were first evaluated by real-time polymerase chain reaction (RT-PCR). The results indicated that CD147 expression was significantly decreased in the CD147si2- and CD147si3-treated groups compared with the CD147si1-treated, control and siRNA NC groups in BT474, SKBR3, MDA-MB453 and HCC1954 cells (Figure 3A, p≤0.01). Western blot analysis also confirmed that CD147si2 and CD147si3 decreased the CD147 protein levels in the four cell lines (Figure 3B). Thus, we chose CD147si2 and CD147si3 to inhibit CD147 expression in further experiments.

**Table 1:** Sequences of the designed CD147-specific siRNAs

siRNA	Sequence
CD147si1	GAGGUGCUGGUGCUGGUCACCAUCA
CD147si2	AGUGAAGGCUGUGAAGUCGUCAGAA
CD147si3	UCCGAGAGCAGGUUCUUCGUGAGUU
siRNA negative control	Stealth siRNA negative control

### CD147 knockdown enhances the inhibitory effect of trastuzumab in HER2-positive cancer cells

**Figure 4:**
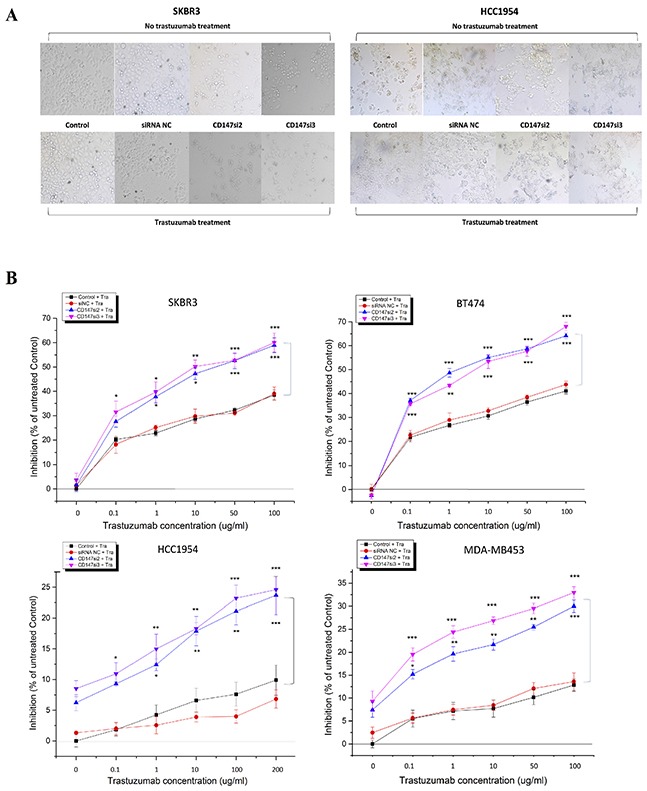
CD147 knockdown decreases cell viability in HER2-positive breast cancer *in vitro* after trastuzumab treatment **A.** Changes in the morphologies and numbers of HER2-positive cancer cells after treatment of SKBR3 and HCC1954 cells with 10 and 200 μg/mL trastuzumab, respectively, for 4 days (100× magnification). Note: a trastuzumab concentration of 0 μg/mL was used for the treatment with CD147 siRNA alone. **B.** Trastuzumab inhibition of cell viability, as measured by CCK-8 assay, occurred in a dose-dependent manner after 4 days of treatment with trastuzumab at five different concentrations. **C.** Trastuzumab inhibits cell viability in a time-dependent manner. The data are presented as the mean ± standard error of the mean (SEM) of three independent experiments. *p<0.05, **p<0.01 and ***p<0.001 vs. controls.

To further understand the role of CD147, we investigated whether the suppression of CD147 expression by two specific siRNAs altered the response of HER2-positive cancer cells to trastuzumab using different concentrations and treatment times. The morphologies and numbers of SKBR3, BT474, HCC1954 and MDA-MB453 cells were altered following trastuzumab treatment for 96 h. Here, we only display the changes in SKBR3 and HCC1954 cells as representatives (Figure 4A). First, we examined the responses of the four cell lines to treatments with different concentrations of trastuzumab for 96 h by CCK-8 assay (Figure 4B) to determine the optimal concentration. Interestingly, at 0 μg/mL trastuzumab, siRNA-mediated silencing of CD147 improved the responses of the resistant cell lines HCC1954 and MDA-MB453 to trastuzumab compared with the control cells (parental and siRNA NC-treated cells), whereas the sensitive cell lines SKBR3 and BT474 displayed the opposite responses, although no significance differences were detected compared with the controls in the four cell lines. However, starting at 0.1 μg/mL trastuzumab, the CD147-knockdown-plus-trastuzumab treatment significantly inhibited cell proliferation in a dose-dependent manner compared with the CD147-knockdown or trastuzumab treatment alone in both the resistant and sensitive cell lines (p<0.05 in SKBR3, HCC1954 and MDA-MB453 cells; p<0.001 in BT474 cells), although the sensitive SKBR3 and BT474 cells maintained strong reactions to trastuzumab treatment alone, while the resistant HCC1954 and MDA-MB453 cells displayed weak reactions There were no significant differences in the treatment responses between the two control groups in the four cell lines. Following treatment with 10 μg/mL trastuzumab, the inhibition rates were approximately 50% in the CD147-knockdown groups of SKBR3 and BT474 cells; therefore, a concentration of 10 μg/mL was used in subsequent experiments, except for the HCC1954 and MDA-MB453 cell treatments, for which 100 μg/mL was used.

Then, time-dependent analysis was performed the four cell lines (Figure 4C). For each treatment time, the trends in cell proliferation were similar to those observed in dose-dependent analysis in the four cell lines. The only difference was that the inhibition rates in the CD147-knockdown groups were markedly increased compared with those in the control groups of BT474 and SKBR3 cells (p<0.001) and HCC1954 cells (p<0.05) following the 3-day treatment, whereas in MDA-MB453 cells, such enhancement was observed following the 4-day treatment (p<0.01). No significant differences in the inhibition rates were noted between the siRNA NC and control groups in the four cell lines.

These results suggest that CD147 knockdown and trastuzumab have an additive effect on inhibition of HER2-positive breast cancer cell viability.

### CD147 knockdown induces HER2-positive cancer cell apoptosis under trastuzumab treatment

**Figure 5:**
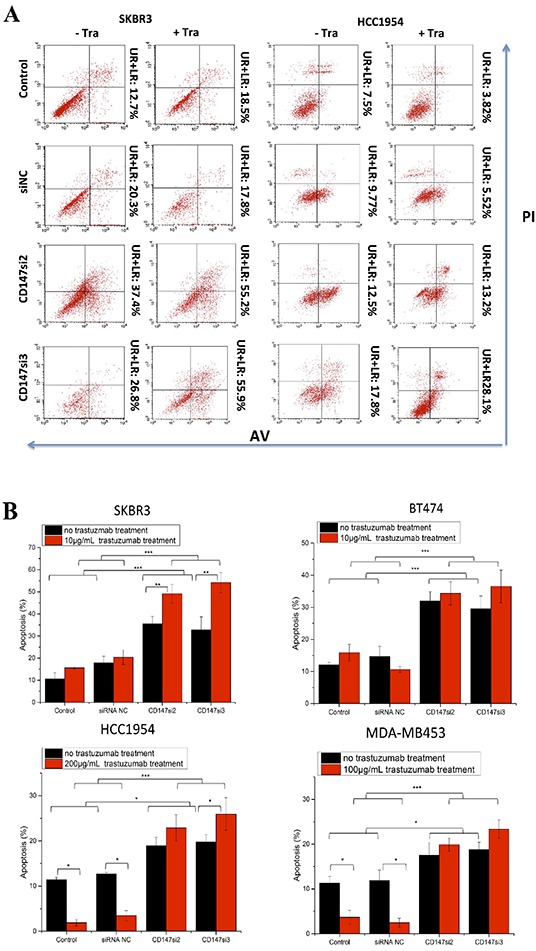

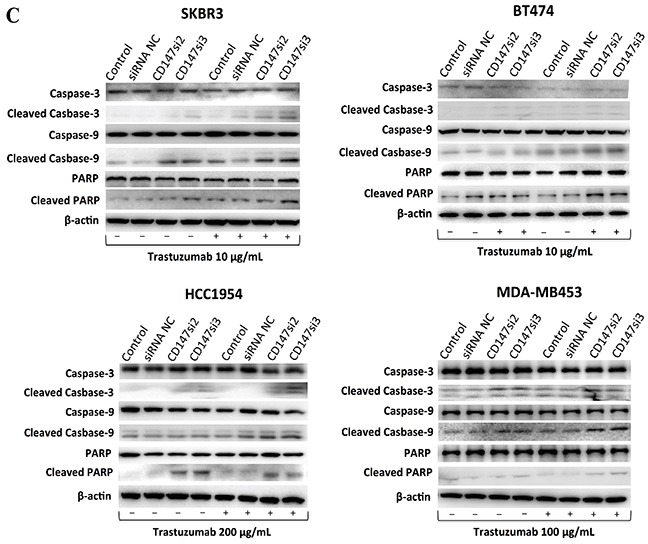
CD147 knockdown alters cell apoptosis in HER2-positive cancer *in vitro* after trastuzumab treatment **A.** Cell apoptosis was measured by FACS after treatment of two HER2-positive cancer cell lines with 10 or 200 μg/mL trastuzumab for two days. For each cell line, no trastuzumab treatment is presented on the left, and trastuzumab treatment is presented on the right. **B.** Apoptosis was altered after the different treatments in the four HER2-positive breast cancer cell lines. **C.** Expression of apoptotic proteins was examined by Western blotting. The third and fourth lanes corresponded to the CD147-siRNA treatment alone. The data are presented as the mean ± SEM of more than three independent experiments. *p<0.05, **p<0.01 and ***p<0.001 vs. controls.

We also assessed apoptosis of SKBR3, BT474, HCC1954 and MDA-MB453 cells following trastuzumab treatment using Annexin V (AV) and propidium iodide (PI) staining. Here, we only present the AV and PI staining of SKBR3 and HCC1954 cells as representatives of sensitive and resistant cells, respectively (Figure 5A). Apoptosis was increased in the CD147-knockdown and CD147-knockdown-plus-trastuzumab groups compared with the control groups in the four cell lines (Figure 5B). Furthermore, only CD147-knockdown treatment notably enhanced apoptosis compared with the control groups especially in SKBR3 and BT474 cells. Under trastuzumab treatment alone, apoptosis was unpredictably strongly increased in the sensitive SKBR3 and BT474 cells, whereas it was markedly decreased in the resistant HCC1954 (p<0.05) and MDA-MB453 (p<0.05) cells compared with control cells. Moreover, compared with CD147-knockdown treatment alone, CD147-knockdown-plus-trastuzumab treatment increased apoptosis in SKBR3 (p<0.01) and HCC1954 (p<0.05) cells, and this result was not observed in BT474 or MDA-MB453 cells. However, compared with trastuzumab treatment alone, CD147-knockdown-plus-trastuzumab treatment significantly increased apoptosis in the four cell lines. These findings indicated that inhibition of CD147 resulted in high rates of apoptosis in SKBR3 and BT474 cells and especially in trastuzumab-resistant HCC1954 and MDA-MB453 cells, which might be the primary reason that CD147 knockdown improved the efficacy of trastuzumab. No significant differences in apoptosis were noted between the siRNA NC and parental cell groups in the four cell lines.

Furthermore, we detected the levels of apoptotic proteins in the four cell lines (Figure 5C). Consistent with the above results, cleaved Caspase-3/9 and cleaved PARP were increased only in the CD147-knockdown and CD147-knockdown-plus-trastuzumab treatment groups compared with the control groups, regardless of trastuzumab treatment. In particular, the levels of cleaved Caspase-3/9 were markedly altered in SKBR3 cells, in addition to the levels of cleaved Caspase-9 and PARP in BT474 and MDA-MB453 cells and those of cleaved Caspase-3 and PARP in HCC1954 cells. There were no significant differences in the levels of apoptotic proteins between the two control groups in the four cell lines.

### Inhibition of CD147 decreases MAPK and/or Akt phosphorylation during trastuzumab treatment in different HER2-positive breast cancer cells

**Figure 6:**
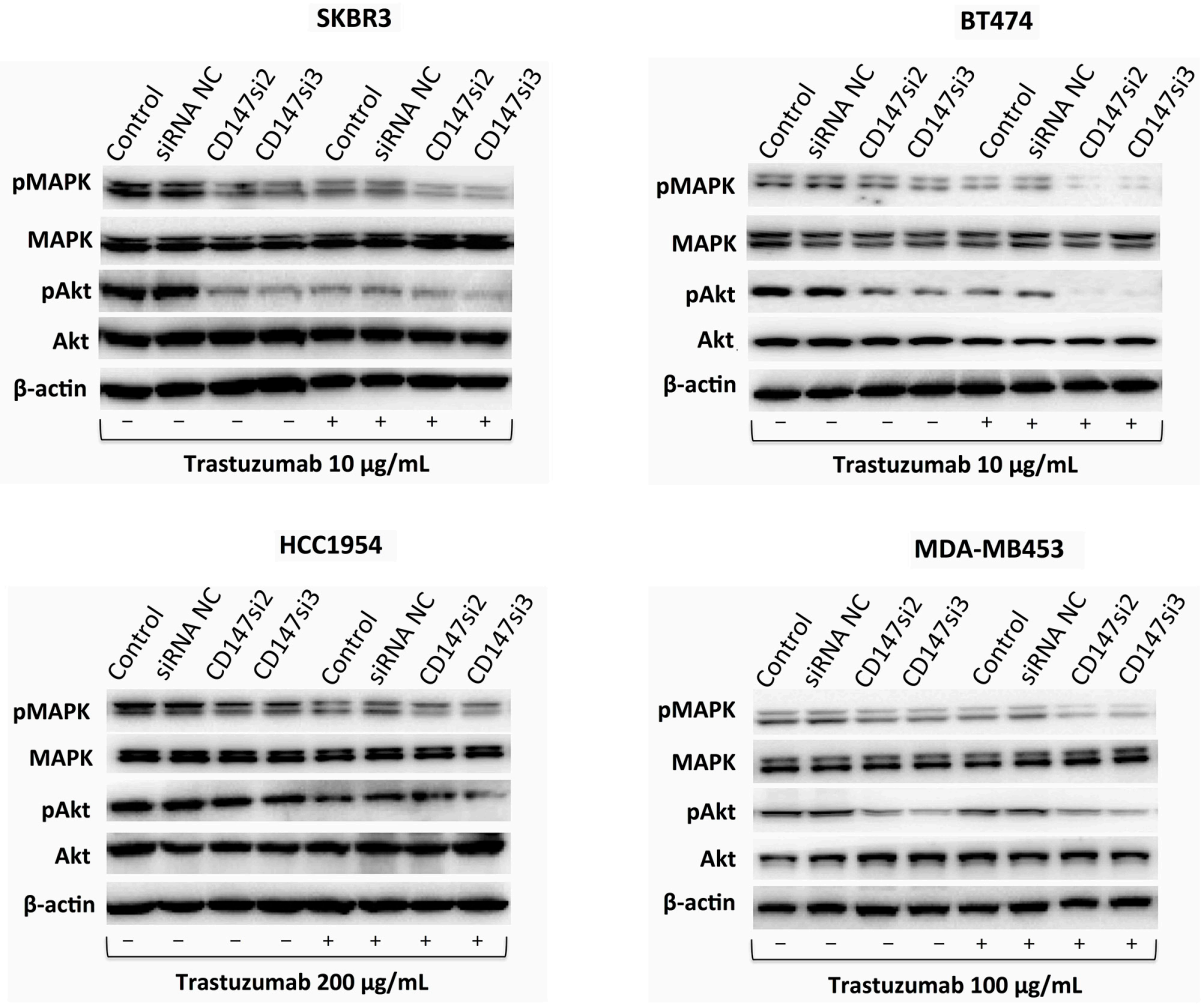
Changes in signaling pathways in different HER2-positive cancer cell lines The MAPK and Akt pathways and their phosphorylation patterns, as assessed by Western blotting, following trastuzumab treatment for 1 h. The data are presented as the mean of three independent experiments.

The MAPK/Erk or PI3K/Akt pathway is the primary downstream signaling pathway inhibited by trastuzumab in HER2-positive cancer cells [4]. Thus, we examined changes in the phosphorylation of MAPK and Akt before and after trastuzumab treatment for 1 h in SKBR3, BT474, HCC1954 and MDA-MB453 cells. As shown in Figure 6, CD147-knockdown or trastuzumab treatment alone decreased MAPK phosphorylation compared with the untreated control groups in the four cell lines. Moreover, CD147-knockdown-plus-trastuzumab treatment dramatically decreased MAPK phosphorylation compared with CD147-knockdown or trastuzumab treatment alone. No significant differences were observed between the siRNA NC and parental cell groups in the four cell lines with or without trastuzumab treatment.

The same decreasing trend in Akt phosphorylation was observed in BT474, SKBR3 and HCC1954 cells regardless of whether CD147-knockdown, trastuzumab or CD147-knockdown-plus-trastuzumab treatment was applied. However, little or no change in Akt phosphorylation was observed in the MDA-MB453 control groups after drug treatment alone; surprisingly, a decrease in Akt phosphorylation was observed following CD147-knockdown or CD147-knockdown-plus-trastuzumab treatment. There were no significant differences in Akt phosphorylation between the two control groups in the four cell lines with or without trastuzumab treatment.

### CD147 knockdown enhances the antitumor efficacy of trastuzumab in a breast cancer xenograft model

**Figure 7:**
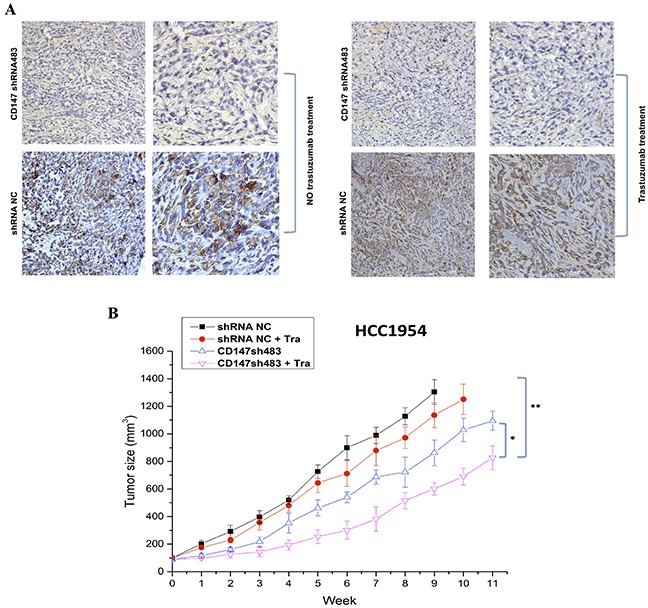
CD147 knockdown inhibits the growth of HER2-positive cancer cells *in vivo* **A.** CD147 expression was decreased in the CD147-knockdown groups with or without trastuzumab treatment, as shown by IHC of mouse tumor tissues. 100× (left) and 400× (right) magnification. **B.** Tumor volumes of HCC1954 breast tumor xenografts after trastuzumab treatment (10 mg/kg). The data are presented as the mean ± SEM of three independent experiments. *p<0.05 and **p<0.01 vs. controls.

The therapeutic efficacy of trastuzumab following CD147 suppression was examined in nude mice bearing established HCC1954-shRNA NC and HCC1954-sh483 xenograft tumors. Here, HCC1954-shRNA NC and HCC1954-sh483 cells were screened from stable CD147 knockdown cell lines established in HCC1954 cells using the shRNA interference method (Supplementary Figure S2). Trastuzumab exhibited increased suppression of tumor growth in the CD147-knockdown group compared the shRNA NC group in the HCC1954 xenograft model (Figure 7). Notably, based on the IHC results in tumor tissues, CD147 expression was suppressed in CD147-knockdown group compared with shRNA NC group (Figure 7A), in accordance with the *in vitro* results (Supplementary Figure S3). Moreover, CD147-knockdown-plus-trastuzumab treatment exhibited greater efficacy in inhibiting HCC1954 tumor growth than trastuzumab or CD147-knockdown treatment alone (Figure 7B). Specifically, compared with no treatment in the shRNA NC group, CD147-knockdown-plus-trastuzumab treatment resulted in 38.5% regression of HCC1954 tumors (p<0.01), and CD147-knockdown treatment alone caused 15% regression (p<0.05), whereas the trastuzumab treatment alone resulted in no significant regression. These results indicated that CD147 knockdown improved the suppressive effect of trastuzumab on tumor growth *in vivo*.

## DISCUSSION

Trastuzumab is widely used to prolong the survival of HER2-positive breast cancer patients, but a low objective response rate and the appearance of resistance have been reported [3, 4]. These findings prompted us to identify novel ways to improve the efficacy of trastuzumab treatment in trastuzumab-responsive and unresponsive patients. CD147 is an important molecule in cancer [21], and different CD147 expression levels may have different clinical significance. Overexpression is always correlated with poor prognosis in certain cancers [24–29]. However, few reports have demonstrated a link between CD147 and trastuzumab treatment in HER2-positive breast cancer cells. Therefore, we first specifically detected CD147 expression and demonstrated that its knockdown improved the efficacy of trastuzumab in HER2-positive cancer cells. Our results were consistent with those of previous studies of CD147 expression in cancer cells using western blotting [30], and serve as a foundation for further analyses of relative CD147 expression. CD147-knockdown-plus-trastuzumab treatment significantly inhibited cell proliferation and induced cell apoptosis *in vitro* and significantly improved the response of HER2-positive breast cancer cells to trastuzumab *in vivo* compared with the controls. These results indicate that inhibition of CD147 may be a new strategy for increasing the efficacy of trastuzumab in the clinical setting.

Next we preliminarily examined how CD147 knockdown improves the efficacy of trastuzumab. One hypothesis involved altered apoptosis of SKBR3, BT474, HCC1954 and MDA-MB453 cells in response to trastuzumab treatment. Caspase-3/9 and PARP cleavages, as well as AV and PI staining, were enhanced in the CD147-knockdown groups compared with the control groups, indicating that CD147 has an important role in tumor cell apoptosis. CD147 has been reported to play similar roles in HCC [10, 31–33], head and neck squamous cell carcinomas [34], non-small cell lung carcinoma (NSCLC) [35], and ovarian cancer [36]. Following CD147 knockdown, Caspase-3 is activated in HCC cells [10]; further, increased Caspase-3 and PARP cleavages occur in human leukemic monocyte lymphoma U937 cells [37], whereas the addition of CD147 to melanoma cell lines has been shown to result in a decrease in Caspase-3/7 activity [38]. However, the effect of trastuzumab on cell apoptosis has not been well studied in recent years. Here, we determined the rates of trastuzumab-induced apoptosis in four parental HER2-positive cancer cell lines. We found that trastuzumab-induced apoptosis was decreased in the parental SKBR3 and BT474 cells, similar to previous studies [39, 40], and it was even decreased in the parental HCC1954 and MDA-MB453 cells, which again may demonstrate that these two cell lines are trastuzumab resistant.

The mechanism underlying the effect of trastuzumab treatment on HER2-positive cancer cells primarily involves the MAPK and Akt pathways [4, 7, 8, 41]. CD147 is involved in HCC chemoresistance through the MAPK/Erk signaling pathway, and it activates PI3K/Akt signaling to promote tumor invasion and metastasis [17]. Therefore, we also preliminarily examined changes in MAPK and Akt phosphorylation after CD147 knockdown in the presence or absence of trastuzumab treatment. According to our results, trastuzumab treatment in the control groups decreased MAPK (Thr202/Tyr204) and Akt (Ser473) phosphorylation in SKBR3, BT474 and HCC1954 cells and reduced MAPK phosphorylation in MDA-MB453 cells (which harbor a mutated Akt gene). CD147-knockdown or CD147-knockdown-plus-trastuzumab treatment reduced MAPK and Akt phosphorylation in all four cell lines compared with the controls, indicating that CD147 knockdown might increase the effects of trastuzumab on the MAPK and Akt pathways in HER2-positive breast cancer cells, and the effects of CD147 knockdown and trastuzumab were overlapping. However, further studies are needed to determine the exact mechanism underlying these effects.

CD147 may be clinically significant not only as a marker of activated regulatory T cells but also as a potential diagnostic marker of early-stage disease [21] or a prognostic marker, as reported in endometrial cancer [24], gastric cancer [25], HCC [27], lupus nephritis [42] and UCB [28]. CD147 is also an indicator of five-year survival rate in NSCLC [29]. Another study has demonstrated that CD147 expression is associated with poor overall survival in patients with glioblastoma and has suggested that it may be used to predict the treatment response of glioblastoma patients [26]. CD147 expression is correlated with axillary lymph node involvement, TNM (tumor, node, metastasis) staging and breast cancer HER2 expression based on S-P IHC analysis (p<0.01, p<0.05 and p<0.01, respectively) [22]. Our study demonstrated a relationship between CD147 and antibody drug treatment efficacy, but further study of the relationship between CD147 and HER2 is needed. According to our findings, CD147 could serve as a therapeutic target and a potential indicator of the response to trastuzumab treatment and prognosis of HER2-positive breast cancer patients. However, it remains unclear whether high CD147 expression in HER2-positive breast cancer is related to a poor response to antibody drug treatment, and further clinical investigations are needed to confirm this association. The finding that CD147 knockdown may improve antitumor efficacy in patients with HER2-positive solid tumors provides a new strategy for constructing bispecific antibodies against CD147 and HER2 or adding other effective targets, which will be extremely significant for clinical tumor immunotherapy in the future.

## MATERIALS AND METHODS

### Cell lines and culture

The human breast cancer cell lines BT474, SKBR-3, MDA-MB453, HCC1954 and JIMT-1 were purchased from the American Type Culture Collection (ATCC). All cell lines were identified by GENEWIZ, Inc. (Beijing, China) and were maintained in DMEM containing 10% fetal bovine serum (FBS), except for HCC1954, which was maintained in RPMI 1640 containing 10% FBS at 37°C in a humidified atmosphere with 5% CO_2_/95% air.

### Western blot analysis

Cells were harvested in RIPA buffer (Beyotime) and maintained on ice. After vortexing for 30 sec, the lysates were cleared by centrifugation at 14,000 g for 5 min. The whole-cell protein extracts were analyzed by Western blotting with the following primary antibodies: CD147 and β-actin (Abcam), p44/42 MAPK, Akt, phosphorylated p44/42 MAPK (Erk1/2) (p-MAPK, Thr202/Tyr204), phosphorylated Akt (p-Akt, Ser473), Caspase-3, Caspase-9, cleaved Caspase-3 (Asp175) and 9 (Asp330) and PARP (Asp214) (Cell Signaling Technology).

### Determination of cell viability via CCK-8 assay

Cells were seeded into 96-well plates at a density of 5.0×10^3^ cells per well and cultured for 24 h. The cells were treated with Stealth siRNAs for 72 h in the knockdown groups and/or with different concentrations of trastuzumab (Roche Ltd.) for 96 h or the otherwise indicated duration. The cells were then incubated with 10 μL CCK-8 solution for 1.5 h at 37°C. Absorbance was detected at 450 nm using a Microplate Reader (Bio-Rad Model 680). The cell survival index was calculated as [A490(trastuzumab+)/A490(trastuzumab−)] · 100%.

### Determination of cell apoptosis via AV staining

Control cells or cells treated with 10 μg/mL trastuzumab were collected and stained with AV-phycoerythrin and PI (Becton Dickinson). Apoptotic cell death was measured by counting the number of cells that stained positive for AV-phycoerythrin, as determined by FACS.

### Immunohistochemistry

For 3,3-diaminobenzidine staining, paraffin-embedded tissue sections were deparaffinized and stained with antibodies against CD147 and HER2 and then with biotin-conjugated secondary antibodies (1:1000), followed by incubation with HRP-conjugated streptavidin (Dianova). The sections were finally counterstained with hematoxylin. The number of tumors in each group was quantified in at least 10 random areas per section. Images were obtained with an OLYMPUS BX51.

### *In vivo* therapeutic analysis

HCC1954-shRNA NC or HCC1954-sh483 cells (5 × 10^6^ per mouse) were subcutaneously inoculated into the mammary fat pads of female BALB/c nude mice. When the tumor volumes reached an average of approximately 100 mm^3^, the mice were randomly divided into groups of 10 mice each. They were then intravenously injected with control human IgG or trastuzumab (10 mg/kg) twice weekly. Tumors were measured with digital calipers, and tumor volumes were calculated using the formula volume = [length × (width)]^2^ ÷ 2.

### Statistical analysis

Experiments were repeated at least three times. Data were analyzed by one-way analysis of variance (ANOVA) or Student's t-test. A p-value of <0.05 was considered significant.

## SUPPLEMENTARY MATERIALS AND METHODS


