# Supplementary material for: CD147 knockdown improves the antitumor efficacy of trastuzumab in HER2-positive breast cancer cells
Source: Oncotarget. 2016 Jun 23;7(36):57737–51. doi: 10.18632/oncotarget.10252 (PMC5295386; doi:10.18632/oncotarget.10252)
Supplement: Supplementary file 1 [file oncotarget-07-57737-s001.pdf]

SUPPLEMENTARY MATERIALS AND METHODS

Treatment with siRNA

SK-BR3, BT474, HCC1954 and MDA-MB453 cells were trypsinized and plated in 6-well culture dishes. At 50% confluence, the cells were transfected with the annealed siRNA-CD147 or with stealth siRNA negative control as a negative control of gene silencing (final concentration, 80 nmol/L), using the Lipofectamine MAX reagent (Invitrogen). The cells were cultured with siRNAs for 72 h.

Reverse transcription and quantitative realtime-PCR

Total RNA was extracted from cells using Trizol (Invitrogen). Total RNA was reverse-transcribed to total cDNA using the GoScript™ Reverse Transcription reagent kit (Promega). The resulting cDNA (0.1 µg) was subjected to real-time PCR amplification using a LightCycler 480 SYBR Green I Master (Roche). The 20-µL reaction mixture contained 2 µL of cDNA template, 10 µL of 2×SYBR Green Mix and 1 mM of each primer for CD147 (GACGACCAGTGGGGAGAGTA; GCGAGGAACT CACGAAGAAC), which generated a 256-bp fragment, or for β-actin (AGCGAGCATCCCCCAAAGTT; GGGC ACGAAGGCTCATCAT T), which generated a 286-bp fragment. β-actin served as an internal control. The amplification cycles were as follows: 94°C for 3 min; 30 cycles at 94°C for 1 min, 56°C for 1 min, and 72°C for 1.5 min; and 72°C for 15 min.

Flow cytometry analysis

Cells (1×10^6 cells/sample) were washed thrice with phosphate-buffered saline (PBS) and resuspended in 100 µL of PBS. The cells were then incubated with 5 µL of 10 µg/mL anti-CD147 antibody and/or anti-HER2 antibody, as well as IgG 1 Isotype control (BD PharMingen), at room temperature for 15 min in the dark. Cells were washed twice with PBS and resuspension in 500 µL of PBS, and cell-surface fluorescence intensity was analyzed using a FACS scan (BD FACSCalibur) and FlowJo software (Tree Star, Inc., CA, USA).

SUPPLEMENTARY FIGURES

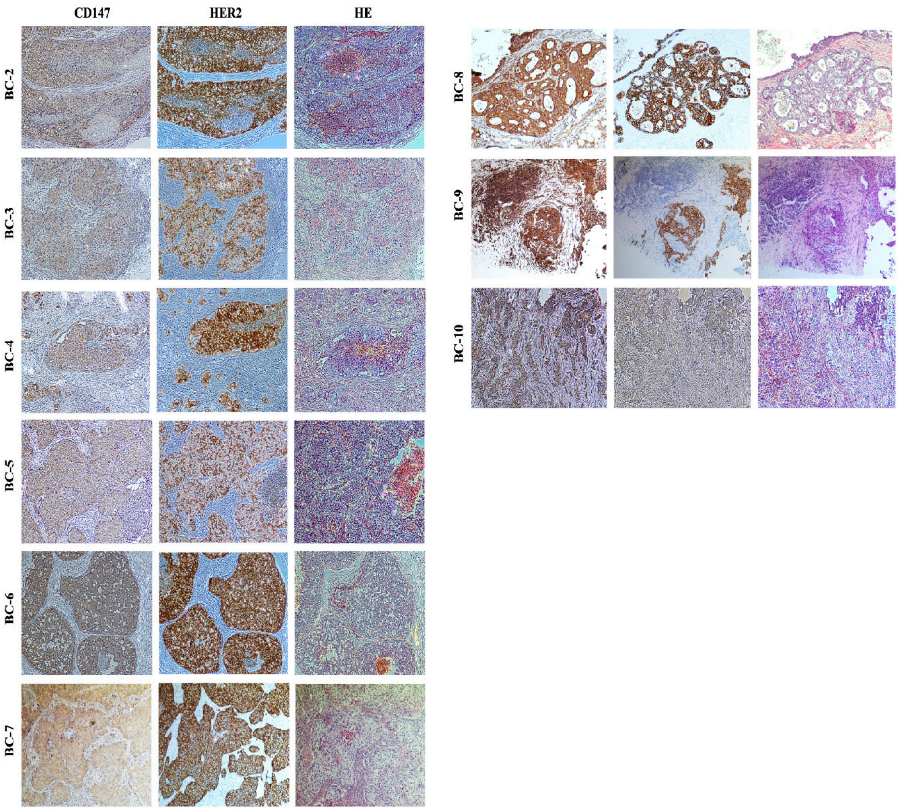

Supplementary Figure S1: Immunohistochemical observation of CD147 and HER2 expression in HER2-positive breast cancer. The first, second and third rank was CD147, HER2 and HE staining, respectively. 100× magnification.

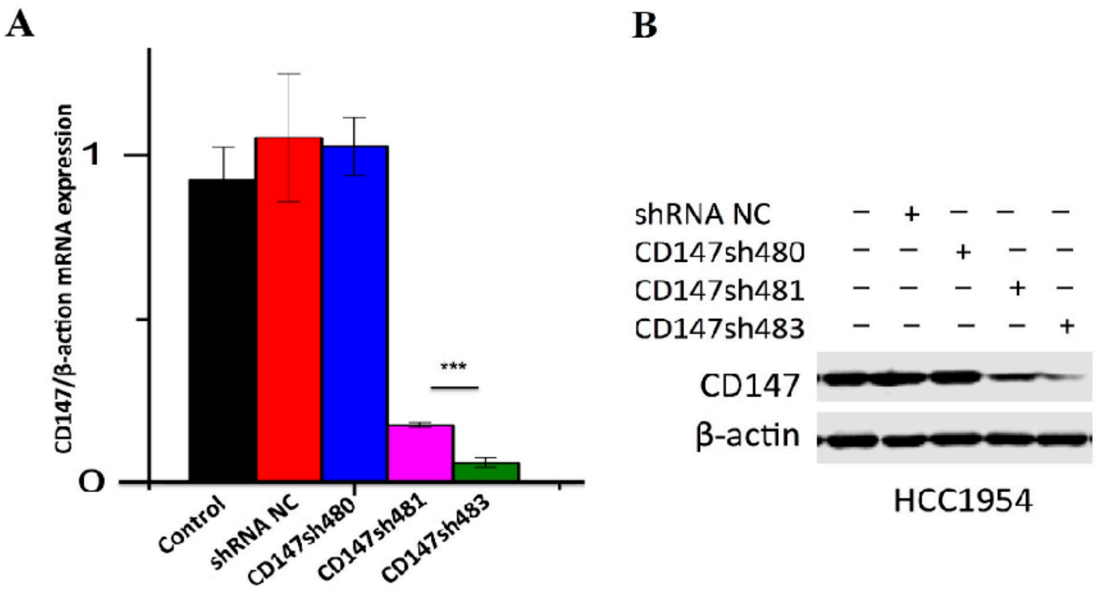

Supplementary Figure S2: CD147 mRNA and protein expression levels were reduced by specific shRNAs. A. CD147 mRNA expression was detected by real-time PCR in different cell lines; β-actin was used as a normalization control. Inhibition of greater than 80% was regarded as effective shRNA interference. *p<0.05, **p<0.01 and ***p<0.001 compared with control. **B.** CD147 protein expression was detected by Western blotting in different cancer cells.

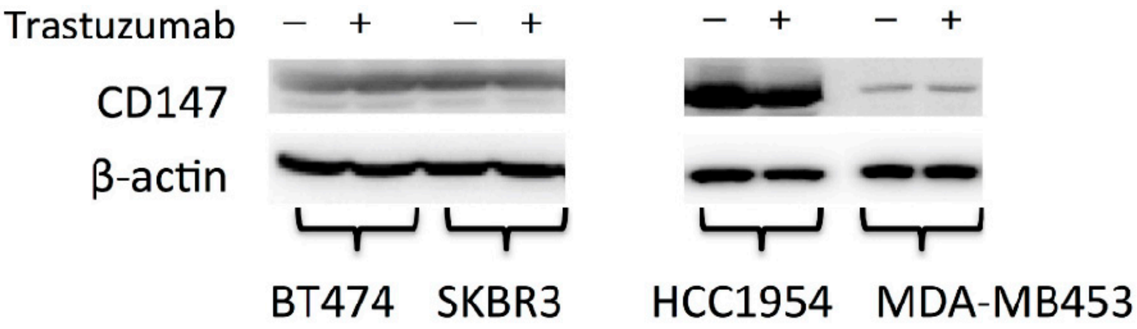

Supplementary Figure S3: CD147 protein expression in HER2-positve breast cancer cells with or without trastuzumab treatment. The data are presented as the mean of three independent experiments.
